# HAT_2_CH_2_ Score Predicts Systemic Thromboembolic Events in Elderly After Cardiac Electronic Device Implantation

**DOI:** 10.3389/fmed.2021.786779

**Published:** 2021-12-24

**Authors:** Ju-Yi Chen, Tse-Wei Chen, Wei-Da Lu

**Affiliations:** Department of Internal Medicine, National Cheng Kung University Hospital, College of Medicine, National Cheng Kung University, Tainan, Taiwan

**Keywords:** atrial high-rate episodes, cardiac implantable electronic device, elderly, HAT_2_CH_2_ score, neurologic events (NE), transient ischemic attacks (TIA)

## Abstract

**Background:** The HAT_2_CH_2_ score has been evaluated for predicting new onset atrial fibrillation, but never for adverse systemic thromboembolic events (STE) in elderly. We aimed to evaluate the HAT_2_CH_2_ score and comparing to atrial high rate episodes (AHRE) ≥24 h for predicting STE in older patients with cardiac implantable electronic devices (CIED) implantation.

**Methods:** We retrospective enrolled 219 consecutive patients ≥ 65 years of age undergoing CIED implantation. The primary endpoint was subsequent STE. For all patients in the cohort, the CHA_2_DS_2_-VASc, C_2_HEST, mC_2_HEST, HAVOC, HAT_2_CH_2_ scores and AHRE ≥ 24 h were determined. AHRE was defined as > 175 bpm lasting ≥ 30 s. Multivariate Cox regression analysis with time-dependent covariates was used to determine variables associated with independent risk of STE.

**Results:** The median patient age was 77 years, and 61.2% of the cohort was male. During follow-up (median, 35 months), 16 STE occurred (incidence rate, 2.51/100 patient-years; 95% CI, 1.65–5.48). Multiple Cox regression analysis showed that the HAT_2_CH_2_ score (HR, 3.405; 95% CI, 2.272–5.104; *p* < 0.001) was an independent predictor for STE. The optimal HAT_2_CH_2_ score cutoff value was 3, with the highest Youden index (AUC, 0.907; 95% CI, 0.853–0.962; *p* < 0.001). The STE rate increased with increasing HAT_2_CH_2_ score (*p* < 0.001).

**Conclusions:** This study is the first to show the prognostic value of the HAT_2_CH_2_ score for STE occurrence in older patients with CIEDs.

## Key Points

- The HAT_2_CH_2_ score predicts STE in older patients with CIED and without prior atrial fibrillation.
- HAT_2_CH_2_ score of 0–2 indicates low-risk, 3–5: medium-risk, and 6–7: high-risk, for STE events.

## Why Does This Paper Matter?

Our study shows the prognostic value of HAT_2_CH_2_ score for STE occurrence in older patients with CIEDs.

## Introduction

A variety of cardiac implantable electronic devices (CIED) are used in the elderly, including permanent pacemakers (PPM) (1), cardiac resynchronization therapy (CRT) (2), and implantable cardioverter defibrillators (ICD) (2). Atrial high-rate episodes (AHRE), commonly detected by CIED, are an important risk factor for new-onset atrial fibrillation (3) The latest European Society of Cardiology guidelines (3) regarding non-valvular atrial fibrillation (AF) state that CIED-detected AHRE > 5–6 min and > 180 bpm increase the risk for systemic thromboembolic events (STE). They recommend that AHRE ≥ 24 h should be closely monitored and treated. The CHA_2_DS_2_-VASc score is used for risk stratification of STE (4); however, its utility in non-valvular AF patients is controversial, primarily because this vascular scoring system does not include AF-related parameters (4). One meta-analysis showed that the discrimination power of the CHA_2_DS_2_-VASc score in predicting STE is modest and is similar in the presence or absence of non-valvular AF (4). Thus, more accurate STE prediction models are needed for assessing non-valvular AF and older patients with CIEDs.

The HAT_2_CH_2_ score, based on patient age and the presence of hypertension, stroke or transient ischemic attack, chronic obstructive pulmonary disease (COPD), and heart failure, was developed in 2010 for identifying patients who are likely to progress to sustained forms of AF in the near future (5). Studies have investigated the use of HAT_2_CH_2_ scores for predicting AF in cancer patients (6), in patients after coronary bypass surgery (7), and emergency-department patients (8). Other scoring systems for predicting new AF, including C_2_HEST (9), mC_2_HEST (10), and HAVOC (11, 12), have demonstrated acceptable discriminating power. For older patients with CIEDs, a small number of studies have investigated the performance of the HAT_2_CH_2_, C_2_HEST, mC_2_HEST, and HAVOC scoring systems for predicting new-onset atrial fibrillation and STE.

The present study aims to determine the performance of HAT_2_CH_2_ score for predicting STE and to compare this performance to that of AHRE ≥ 24 h and other scoring systems (CHA_2_DS_2_-VASc, C_2_HEST, mC_2_HEST, and HAVOC) in older patients with CIEDs and no history of AF.

## Methods

Consecutive patients ≥18 years of age who underwent CIED implantation (Medtronic: dual chamber PPM, dual chamber ICD, CRTP, or CRTD) in the Cardiology Department of National Cheng Kung University Hospital from January 2015 to April 2021 were retrospectively included.

### Ethical Considerations

The protocol for this cohort study was reviewed and approved by the Ethics Committee of National Cheng Kung University Hospital and conducted according to guidelines of the International Conference on Harmonization for Good Clinical Practice (B-ER-108-278). All included patients provided signed informed consent for data to be used for later publication at the time of the implantation procedures.

### Data Collection and Definitions

Patient medical histories and data regarding co-morbidities and echocardiographic parameters were collected from medical records for retrospective evaluation. Diabetes mellitus was defined as the presence of symptoms and casual plasma glucose concentration ≥ 200 mg/dL, fasting plasma glucose concentration ≥ 126 mg/dL, 2-h plasma glucose concentration ≥ 200 mg/dL from a 75-g oral glucose tolerance test, or taking medication for diabetes mellitus. Hypertension was defined as in-office systolic blood pressure ≥ 140 mmHg and/or diastolic blood pressure ≥ 90 mmHg or taking antihypertensive medication. Dyslipidemia was defined as low-density lipoprotein ≥ 140 mg/dL, high-density lipoprotein <40 mg/dL, triglycerides ≥ 150 mg/dL, or taking medication for dyslipidemia. Chronic kidney disease (CKD) was defined as an estimated glomerular filtration rate (eGFR) <60 mL/min/1.73 m^2^ for at least 3 months. The primary endpoint for this study was the occurrence of STE after the date of CIED implantation, including stroke or transient ischemic attack (TIA) diagnosed by experienced neurologists based on clinical symptoms and brain imaging; pulmonary embolism diagnosed by experienced cardiologists based on clinical symptoms and chest imaging. TIA was defined as a transient episode of neurological dysfunction caused by focal brain, spinal cord, or retinal ischemia, without acute infarction. Pulmonary embolism was diagnosed using computed tomography pulmonary angiography or pulmonary angiography. For each outcome, only the first event of that outcome in a given patient was included. For the composite outcome, only the first event in a given patient was included.

AHRE data were extracted from the devices *via* telemetry at each office visit (every 3–6 months). AHRE electrograms were reviewed by at least one experienced electrophysiologist, who carefully considered the possibility that the AHRE included lead noise or artifacts, far-field R-waves, or paroxysmal supraventricular tachycardia and thus visually confirmed AF in the detected AHRE. Atrial sensitivity was programmed to 0.3 mV with bipolar sensing of Medtronic devices. AHRE was defined as heart rate > 175 bpm and at least 30 s of atrial tachyarrhythmia recorded by the device on any day during the study period.

### Scoring System Assessments

CHA_2_DS_2_-Vasc score (4): Range from 0 to 9. History of heart failure, hypertension, diabetes, vascular disease, age 65–74 years, and female sex each is calculated as 1 point; 75 years or older and prior stroke, TIA, or thromboembolism each is calculated as 2 points; C_2_HEST score (9): Range from 0 to 8. C_2_: CAD/COPD (1 point each); H: hypertension (1 point); E: elderly (age ≥ 75 years, 2 points); S: systolic HF (2 points); and T: thyroid disease (hyperthyroidism, 1 point); mC_2_HEST score (10): Range from 0 to 8. C_2_: CAD/COPD (1 point each); H: hypertension (1 point); E: elderly (age 65–74 years, 1 point; age ≥ 75 years, 2 points); S: systolic HF (2 points); and T: thyroid disease (hyperthyroidism, 1 point); HAVOC score (11): H: hypertension (2 points); A: age (age ≥ 75 years, 2 points); V: valvular heart disease (2 points), peripheral vascular disease (1 point); O: obesity (1 point); C: congestive heart failure (4 points) and coronary artery disease (2 points). HAT_2_CH_2_ score (5): Range from 0 to 7. Hypertension, 1 point; age >75 years, 1 point; stroke or transient ischemic attack, 2 points; chronic obstructive pulmonary disease (COPD), 1 point; and heart failure, 2 points.

### Statistical Analysis

Categorical variables are presented as percentages, and continuous variables are presented as the mean and standard deviation for normally distributed values or medians and as interquartile interval (IQI) for non-normally distributed values. Normal distribution for continuous variables was assessed using the Kolmogorov–Smirnov method. Pearson's chi-square test or Fisher's exact-test was used to determine differences in baseline characteristics for categorical variables, and a two-sample Student's *t*-test or Mann–Whitney *U*-test was used to analyze continuous variables. Survival was estimated using the Kaplan–Meier method, and differences in survival were evaluated using the log-rank test. Multivariate Cox regression analysis was used to identify variables associated with STE occurrence, reported as hazard ratios (HR) with 95% confidence intervals (CI). Parameters with *p* < 0.05 in univariable analysis and age, gender, body mass index, were entered into multivariable analysis. The receiver-operating characteristic (ROC) area under the curve (AUC) of the HAT_2_CH_2_ score and the associated 95% confidence interval (CI) was evaluated for association with future STE after CIED implantation. The optimal cutoff values with the highest Youden index were chosen based on the results of ROC curve analysis and used to evaluate the associated values of the HAT_2_CH_2_ score for determining STE. For all comparisons, *p* < 0.05 was considered statistically significant. All data were analyzed using SPSS statistical package version 23.0 (SPSS Inc. Chicago, IL, USA).

## Results

Between January 1, 2014 and April, 2021, a total of 453 consecutive patients who underwent Medtronic CIED implantation at National Cheng Kung University Hospital were recruited. Patients with previous AF (*n* = 105) and those age <65 years (*n* = 129) were excluded. The final analysis included 219 patients, of which 16 had experienced STE.

The median follow-up period was 35 months after CIED implantation. Table 1 shows patient baseline demographic and clinical characteristics according to the presence or absence of STE. The median age was 77 years, and 61.2% were men. Most patients were not obese. The types of CIED included dual chamber PPM (173; 79.0%), dual chamber ICD (66; 21.0%); CRTP (23; 7.3%); and CRTD (5; 1.6%). The most common indication for CIED implantation was sick sinus syndrome (53.9%), followed by atrioventricular block (25.1%) (Table 1). We observed an overall atrial pacing median of 33.5% and ventricular pacing median of 4.3%. High percentages of hypertension (92.2%), hyperlipidemia (86.3%), diabetes (52.1%), and CKD (39.7%) suggest a relatively high risk of STE for the entire study cohort. Ninety-two patients (42.0%) received antiplatelet agents and 21 patients (9.6%) received anticoagulants. Data regarding the type and incidence of STEs are reported in Table 2. The total number of STEs was 16 [incidence rate (IR), 2.51/100 patient-years; 95% CI, 1.65–5.48] (Table 2).

**Table 1:** Baseline characteristics of the overall study group and with/without systemic thromboembolic events.

	**All patients (*N* = 219)**	**Systemic thromboembolic event**		**Univariate *p***	**Multivariate Cox regression analysis**		
		**Yes (*N* = 16)**	**No (*N* = 203)**		**HR**	**95%CI**	***p***
Age (years)	77 (71–84)	78 (70–84)	77 (71–84)	0.905	0.910	0.824–1.006	0.065
					**0.909**	**0.822–1.006**	**0.064**
**Sex**				0.112	1.302	0.317–5.346	0.715
					**1.217**	**0.287–5.167**	**0.790**
Male	134 (61.2%)	13 (81.3%)	121 (59.6%)				
Female	85 (38.8%)	3 (18.8%)	82 (40.4%)				
BMI (kg/m^2^)	24.2 (22.2–25.9)	23.9 (22.7–27.0)	24.3 (22.1–25.9)	0.746	1.041	0.832–1.302	0.726
					**1.038**	**0.836–1.288**	**0.738**
**Device type**				0.437			
Dual chamber PM	173 (79.0%)	15 (93.8%)	158 (77.8%)				
Dual chamber ICD	23 (10.5%)	0 (0.0%)	23 (11.3%)				
CRTP	19 (8.7%)	1 (6.3%)	18 (8.9%)				
CRTD	4 (1.8%)	0 (0.0%)	4 (2.0%)				
**Primary indication**				0.282			
Sinus node dysfunction	118 (53.9%)	12 (75.0%)	106 (52.2%)				
Atrioventricular block	55 (25.1%)	3 (18.8%)	52 (25.6%)				
Heart failure/VT/VF	46 (21.0%)	1 (6.3%)	45 (22.1%)				
Atrial pacing (%)	33.5 (9.1–79.1)	15.9 (1.1–85.3)	34.3 (10.1–77.7)	0.428			
Ventricular pacing (%)	4.3 (0.2–98.7)	21.9 (0.5–90.9)	2.9 (0.2–98.7)	0.483			
Hypertension	202 (92.2%)	16 (100.0%)	186 (91.6%)	0.620			
Diabetes mellitus	114 (52.1%)	12 (75.0%)	102 (50.2%)	0.070			
Hyperlipidemia	189 (86.3%)	16 (100.0%)	173 (85.2%)	0.137			
Chronic obstructive pulmonary disease	14 (6.4%)	1 (6.3%)	13 (6.4%)	1.000			
Prior myocardial infarction	46 (21.0%)	4 (25.0%)	42 (20.7%)	0.750			
Coronary artery disease	67 (30.6%)	6 (37.5%)	61 (30.0%)	0.533			
**Heart failure**				0.259			
Preserved LVEF	24 (11.0%)	2 (12.5%)	22 (10.8%)				
Reduced LVEF	48 (21.9%)	6 (37.5%)	42 (20.7%)				
None	147 (67.1%)	8 (50.0%)	139 (68.5%)				
Chronic kidney disease	87 (39.7%)	8 (50.0%)	79 (38.9%)	0.383			
Chronic liver disease	8 (3.7%)	1 (6.3%)	7 (3.4%)	0.461			
Thyroid disease	16 (7.3%)	1 (6.3%)	15 (7.4%)	1.000			
Peripheral artery disease	4 (1.8%)	1 (6.3%)	3 (1.5%)	0.263			
Valvular heart disease	25 (11.4%)	1 (6.3%)	24 (11.8%)	1.000			
New atrial fibrillation	22 (10.0%)	5 (31.3%)	17 (8.4%)	0.003	**1.545**	**0.467–5.114**	**0.477**
Hemoglobin (mg/dL)	12.0 (10.8–13.0)	11.3 (10.0–12.0)	12.0 (11.0–13.0)	0.115			
Platelet	201 (168–220)	186 (143–233)	203 (172–220)	0.259			
**Echo parameters**							
LVEF (%)	66.7 (54.0–73.0)	58.5 (42.8–70.8)	67.0 (55.0–74.0)	0.164			
Mitral E/e′	11.8 (9.0–14.0)	11.1 (10.0–15.8)	12.0 (9.0–14.0)	0.480			
LA diameter (cm)	3.8 (3.4–4.1)	3.8 (3.6–4.3)	3.8 (3.3–4.1)	0.414			
RV systolic function (s′, m/s)	12.0 (11.0–14.0)	12.0 (10.3–14.0)	12.0 (11.0–14.0)	0.953			
**Drugs prescribed at baseline**							
Antiplatelets	92 (42.0%)	10 (62.5%)	82 (40.4%)	0.085			
Anticoagulants	21 (9.6%)	2 (12.5%)	19 (9.4%)	0.656			
Beta blockers	72 (32.9%)	7 (43.8%)	65 (32.0%)	0.336			
Ivabradine	16 (7.3%)	2 (12.5%)	14 (6.9%)	0.330			
Amiodarone	37 (16.9%)	1 (6.3%)	36 (17.7%)	0.320			
Flecainide	1 (0.5%)	0 (0.0%)	1 (0.5%)	1.000			
Propafenone	8 (3.7%)	1 (6.3%)	7 (3.4%)	0.461			
Digoxin	4 (1.8%)	0 (0.0%)	4 (2.0%)	1.000			
Non-DHP CCBs	6 (2.7%)	0 (0.0%)	6 (3.0%)	1.000			
RAAS inhibitors	104 (47.7%)	5 (31.3%)	99 (49.0%)	0.171			
Diuretics	34 (15.5%)	5 (31.3%)	29 (14.3%)	0.071			
Statins	90 (41.1%)	3 (18.8%)	87 (42.9%)	0.068			
Metformin	38 (17.4%)	1 (6.3%)	37 (18.2%)	0.317			
SGLT2 inhibitors	9 (4.1%)	0 (0.0%)	9 (4.4%)	1.000			
Follow-up duration (months)	35 (16–53)	17.5 (12.0–49.5)	36.0 (16.0–53.0)	0.091			
CHA_2_DS_2_-VASc score	4 (3–4)	4 (3–5)	4 (3–4)	0.097			
C_2_HEST score	3 (3–4)	3 (3–4)	3 (2–4)	0.211			
mC_2_HEST score	3 (3–4)	4 (3–5)	3 (3–4)	0.170			
HAVOC score	4 (4–8)	6 (4–8)	4 (4–7)	0.272			
HAT_2_CH_2_ score	2 (2–3)	4 (3–6)	2 (2–3)	<0.001	3.405	2.272–5.104	<0.001
					**3.363**	**2.253–5.019**	**<0.001**
AHRE ≥ 24 h	27 (12.3%)	5 (31.3%)	22 (10.8%)	0.017	1.229	0.345–4.381	0.750

*Data are presented as the median (interquartile interval) or n (%). Non-parametric continuous variables, as assessed using the Kolmogorov–Smirnov method, were analyzed using the Mann–Whitney U test. Statistical significance is set at p < 0.05*.

*BMI, body mass index; PM, pacemaker; ICD, implantable cardioverter defibrillator; CRTP, cardiac resynchronization therapy pacemaker; CRTD, cardiac resynchronization therapy defibrillator; VT, ventricular tachycardia; VF, ventricular fibrillation; LVEF, left ventricular ejection fraction; LA, left atrium; RV, right ventricle; non-DHP CCBs, non-dihydropyridine calcium channel blockers; RAAS, renin-angiotensin-aldosterone system; SGLT2, sodium glucose co-transporters 2; CHA_2_DS_2_-Vasc score: Range from 0 to 9. History of heart failure, hypertension, diabetes, vascular disease, age 65–74 years, and female sex each is calculated as 1 point; 75 years or older and prior stroke, TIA, or thromboembolism each is calculated as 2 points; C_2_HEST score: Range from 0 to 8. C_2_, CAD/COPD (1 point each); H, hypertension (1 point); E, elderly (age ≥ 75 years, 2 points); S, systolic HF (2 points); and T, thyroid disease (hyperthyroidism, 1 point); mC_2_HEST score: Range from 0 to 8. C_2_, CAD/COPD (1 point each); H, hypertension (1 point); E, elderly (age 65–74 years, 1 point; age ≥ 75 years, 2 points); S, systolic HF (2 points); and T, thyroid disease (hyperthyroidism, 1 point); HAVOC score: H, hypertension (2 points); A, age (age ≥ 75 years, 2 points); V, valvular heart disease (2 points), peripheral vascular disease (1 point); O, obesity (1 point); C, congestive heart failure (4 points) and coronary artery disease (2 points); HAT_2_CH_2_ score: Range from 0 to 7. Hypertension, 1 point; age >75 years, 1 point; stroke or transient ischemic attack, 2 points; chronic obstructive pulmonary disease, 1 point; heart failure, 2 points; AHRE, atrial high-rate episodes*.

**Table 2:** Type and incidence of systemic thromboembolic events.

**Thromboembolic event type**	**Number**	**Incidence rate (100 patient-years)**	**95% CI**
Transient ischemic attack	9	1.41	0.93–3.08
Ischemic stroke	6	0.94	0.62–2.05
Pulmonary embolism	1	0.16	0.10–0.34
Total events	16	2.51	1.65–5.48

*CI, confidence intervals*.

### Univariate Analysis and Multivariate Cox Regression Analysis to Identify Independent Predictors of STE

Univariate analysis revealed that STE occurrence was significantly associated with AHRE ≥ 24 h, new AF, and HAT_2_CH_2_ score (Table 1). Multivariate Cox regression analysis showed that only the HAT_2_CH_2_ score was independently associated with STE (HR, 3.405; 95% CI, 2.272–5.104; *p* < 0.001). Twenty-two patients (10.0%) had new AF. Only the HAT_2_CH_2_ score differed significantly between those with and without new AF (*p* = 0.025) (Supplementary Table 1).

### ROC-AUC Determination of AHRE and HAT_2_CH_2_ Score Cutoff Values for Factors Predictive of Future STE and Survival Analysis

**Figure 1:**
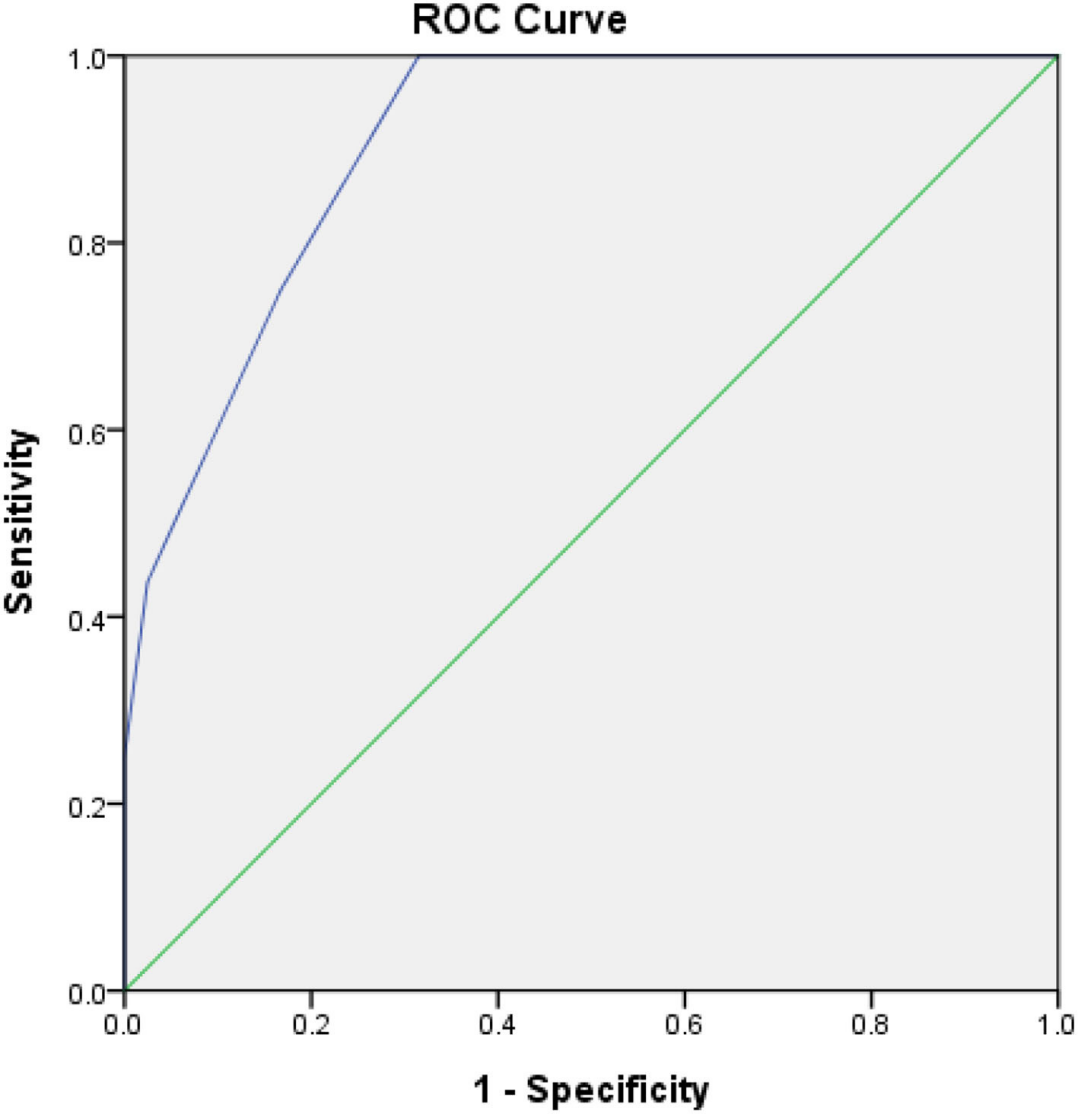
Receiver-operating characteristic curve analysis of the HAT_2_CH_2_ score in patients with CIEDs with subsequent systemic thromboembolic events. HAT_2_CH_2_ score: optimal cutoff value with the highest Youden index, 3; sensitivity, 100.0%; specificity, 80.0%; AUC, 0.907; 95% CI, 0.853–0.962; *p* < 0.001.

**Figure 2:**
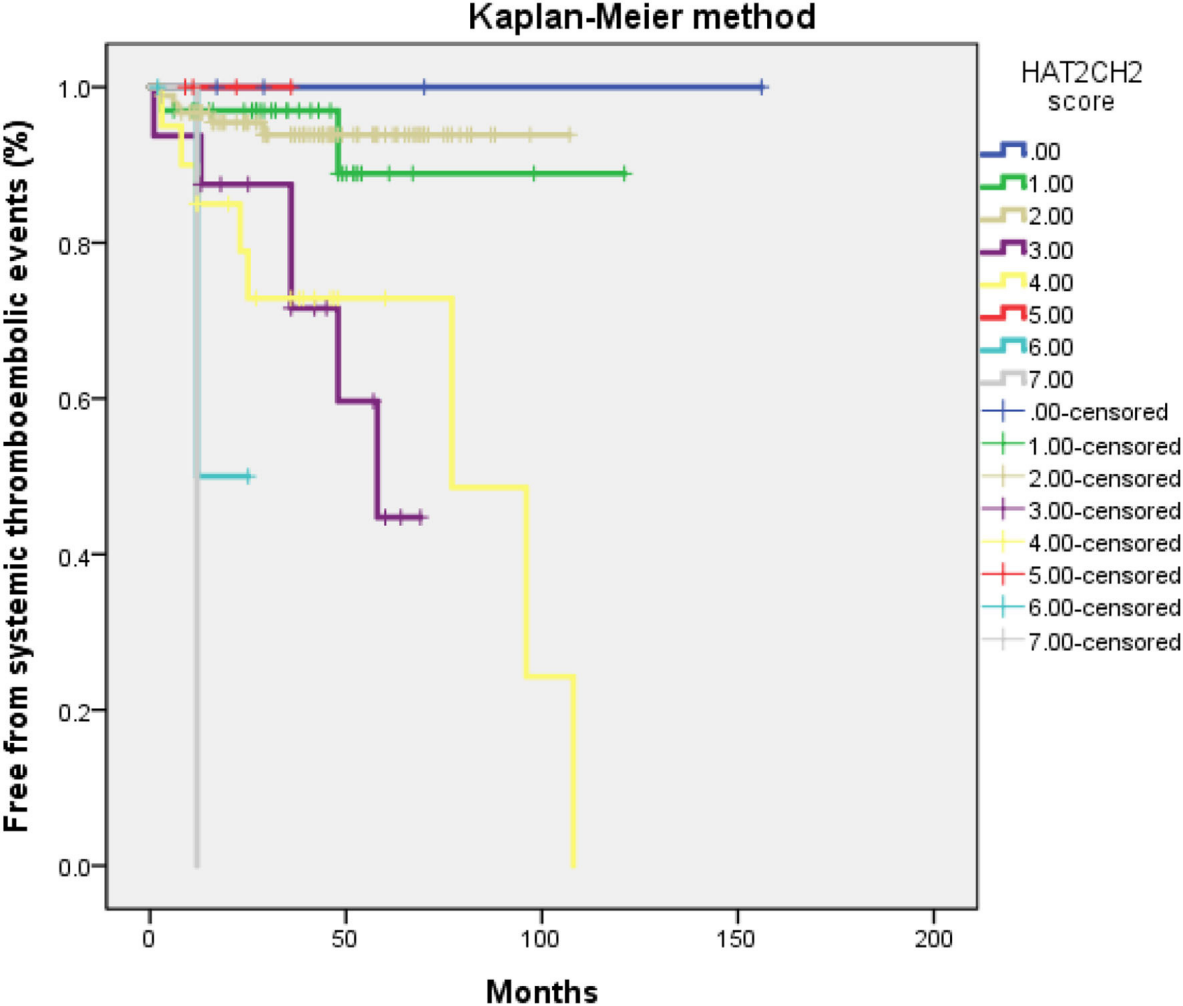
Kaplan–Meier curves depicting the cumulative survival rates free from systemic thromboembolic events with respect to HAT_2_CH_2_ scores (0–7; log-rank *p* < 0.001).

**Figure 3:**
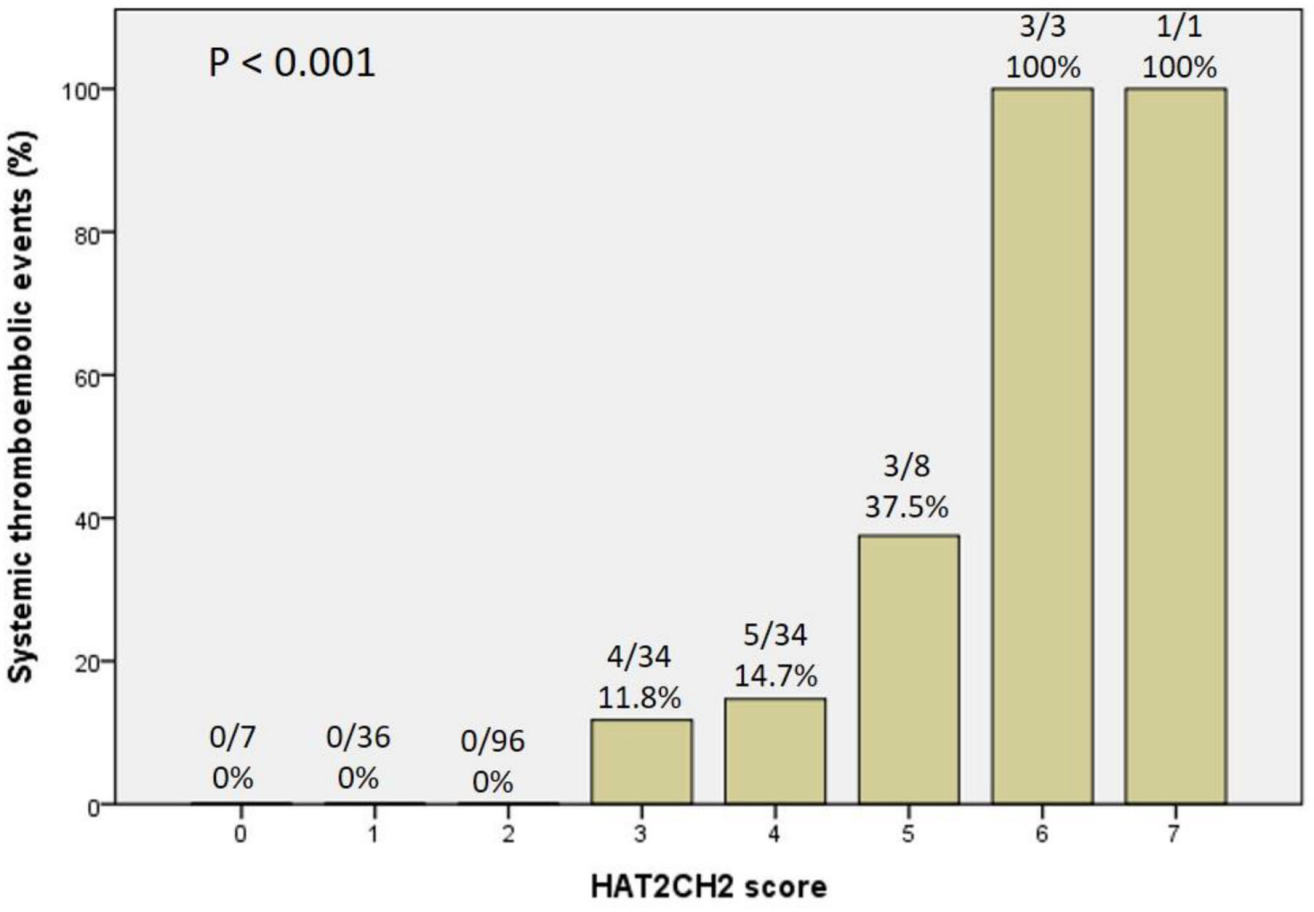
Systemic thromboembolic event rate significantly increased with increasing HAT_2_CH_2_ score.

The optimal HAT_2_CH_2_ score cutoff value predictive of future STE was determined to be 3 according to the highest Youden index (sensitivity, 100.0%; specificity, 80.0%; AUC, 0.907; 95% CI, 0.853–0.962; *p* < 0.001) (see Figure 1). Kaplan–Meier curves depict the cumulative survival rates without STE according to HAT_2_CH_2_ score groups of 0–7. Patients with a HAT_2_CH_2_ score of 4–7 had a higher risk for NE development than did those with a HAT_2_CH_2_ score of 0–3 (log-rank test, *p* < 0.001) (see Figure 2). The rate of NE occurrence significantly increased with increasing HAT_2_CH_2_ score, as follows: 0–2 (0%), 3 (11.8%), 4 (14.7%), 5 (37.5%), to 6–7 (100%) (*p* < 0.001) (see Figure 3). We further classified our study population as very low risk (HAT_2_CH_2_ score, 0–2), low risk (HAT_2_CH_2_ score, 3), medium risk (HAT_2_CH_2_ score, 4), high risk (HAT_2_CH_2_ score, 5), and very high risk (HAT_2_CH_2_ score, 6–7).

## Discussion

The main finding of this study is that the HAT_2_CH_2_ score is significantly and independently associated with STE in a population of older Taiwanese patients with CIED and no history of AF. The optimal HAT_2_CH_2_ score cutoff value for predicting subsequent STE was 3, with 100% sensitivity and 80% specificity. These results suggest that comprehensive assessment of older patients with CIED to determine the HAT_2_CH_2_ score may be warranted to allow for early, aggressive therapy to prevent STE.

The present study was conducted because the performance of several AF-predicting scoring systems (CHA_2_DS_2_-VASc, HAT_2_CH_2_, C_2_HEST, mC_2_HEST, and HAVOC) for predicting subsequent STE in older patients with CIED had not been confirmed. Of these scoring systems, CHA_2_DS_2_-VASc is recommended for predicting the risk of STE in patients with non-valvular AF (3). However, some studies report that the discrimination power of the CHA_2_DS_2_-VASc score for predicting STE in patients without non-valvular AF is insufficient (4, 13, 14). Melgaard et al. (15) showed the predictive accuracy of the CHA_2_DS_2_-VASc score for STE in patients with heart failure and without AF was modest (C-statistic, 0.64; 95% CI, 0.61–0.67). A meta-analysis by Siddiqi et al. (4) including 9 studies of patients (*n* = 453,747) with non-valvular AF and 10 studies of patients (*n* = 138262) without non-valvular AF reports that the discrimination power is modest (C-statistic, 0.67; 95% CI, 0.64–0.70) (4). Furthermore, Hu et al. used data from the Taiwan Health Insurance Research Database, reporting that for patients with venous thromboembolism (*n* = 56 996), the area under the curve of ROC for CHA_2_DS_2_-VASc score for predicting STE was 0.66, which also is modest (13). Therefore, more accurate models are needed for predicting STE in patients without AF.

To the best of our knowledge, the current study is the first to show that the HAT_2_CH_2_ score more accurately predicts the risk of STE in patients with CIED than does the CHA_2_DS_2_-VASc, C_2_HEST, mCHEST, or HAVOC score (Table 1); the AUC of the ROC curve is 0.907, indicating excellent discrimination (Figure 1). We also proposed an STE risk stratification scheme based on the HAT_2_CH_2_ score (0–2, low risk; 3–5, medium risk; and 6–7, high risk) (Figure 3). Validation of this scheme in an external population is needed to confirm the accuracy of HAT_2_CH_2_ scores for predicting STE in patients with CIED.

We also observed that of the scoring systems investigated here, only the HAT_2_CH_2_ score could independently predict new-onset AF in this study population. The HAT_2_CH_2_ score includes COPD as one point instead of diabetes or vascular disease included as one point in the CHA_2_DS_2_-VASc score. Thus, different diseases have different effects on the risk of STE in patients with CIED. For example, systemic inflammation and oxidative stress are associated with COPD and promote platelet hyperactivity and cerebral vascular dysfunction (14), and COPD increases the risk of STE, independent of other shared risk factors of cardiovascular disease (16). Additional prospective studies are needed to elucidate the possible mechanisms underlying COPD-related STE risk and to identify effective preventive interventions.

Current guidelines recommend that patients with AHRE ≥ 24 h should be regarded as having the same risk of STE as those with clinical AF (3). A recent systemic review and meta-analysis also demonstrated that AHRE detected by CIED in patients without prior AF would increase the risks of stroke and clinical AF (17). Therefore, in the current study, we included AHRE ≥ 24 h (Table 1) and new AF (Table 1) in the multivariate Cox regression analysis for predicting STE. However, the HAT_2_CH_2_ score was still the only independent predictor of STE in our cohort. Although the proportion of STE associated with AF increases progressively with age (18), the cause of STE in a large proportion of patients is not cardioembolic. Non-cardioembolic risk factors for STE include atherosclerotic intracranial arterial stenosis (19), carotid atherosclerotic stenosis (20), and complex aortic plaque (21); these conditions share more risk factors with the HAT_2_CH_2_ score parameters than with the AHRE ≥ 24 h or AF. Therefore, the HAT_2_CH_2_ score provides a more comprehensive evaluation of STE risk factors in this population.

### Limitations

The present study has several limitations. First, this was a single-center, retrospective, observational study with a relatively small number of older patients with CIED in a hospital setting, and all patients were Taiwanese. As a result, causality cannot be inferred between the HAT_2_CH_2_ score and STE, and the presence of confounding factors cannot be ruled out. Also, the results may not be generalizable to other populations. Prospective multicenter studies with larger cohorts are required to confirm the results of this study. Second, this study did not investigate the nature of heart rhythms at the time of STE onset. Third, because the patient data were analyzed retrospectively, we could not confirm that patients started anticoagulants for the treatment of CIED-detected AHRE, although these patients were not excluded because no significant difference were found between anticoagulants use and the presence (2, 12.5%) or absence (19, 9.4%) of STE (*p* = 0.656) (Table 1). Fourthly, it is necessary to take into consideration the older patients' heterogeneity in the current retrospective study in terms of frailty and dependence, undergoing a detailed multidimensional geriatric assessment before CIED therapy should be a critically important step. Finally, not all National Institutes of Health Stroke Scale was available in all patients with STE will limit the power to detect the severity of STE.

## Conclusions

STEs are not uncommon in older patients after CIED implantation. The HAT_2_CH_2_ score is an independent predictor of STE risk in this population. Our results suggest that for patients with CIED and no history of AF, determination of the HAT_2_CH_2_ score may be warranted to allow for early, aggressive therapy to prevent STE, especially in those with HAT_2_CH_2_ score ≥ 3.

## Data Availability Statement

The raw data supporting the conclusions of this article will be made available by the authors, without undue reservation.

## Ethics Statement

The studies involving human participants were reviewed and approved by the Ethics Committee of National Cheng Kung University Hospital. All patients provided signed informed consent at the time of CIED implantation for later use of their data in publications.

## Author Contributions

J-YC: conception and design, data analysis and interpretation, statistical analysis, drafting and finalizing the manuscript, and critical revision of the manuscript for important intellectual content. T-WC and W-DL: data acquisition. All authors contributed to the article and approved the submitted version.

## Funding

The authors would like to thank the Ministry of Science and Technology of the Republic of China, Taiwan, for financially supporting this research under contract MOST 110-2218-E-006-017.

## Conflict of Interest

The authors declare that the research was conducted in the absence of any commercial or financial relationships that could be construed as a potential conflict of interest.

## Publisher's Note

All claims expressed in this article are solely those of the authors and do not necessarily represent those of their affiliated organizations, or those of the publisher, the editors and the reviewers. Any product that may be evaluated in this article, or claim that may be made by its manufacturer, is not guaranteed or endorsed by the publisher.

## Abbreviations

AF: atrial fibrillation
AHRE: atrial high-rate episodes
AMI: acute myocardial infarction
CIEDs: cardiac implantable electronic devices
COPD: chronic obstructive pulmonary disease
eGFR: estimated glomerular filtration rate
NE: neurologic events
TIA: transient ischemic attacks.
