# Supplementary material for: HAT2CH2 Score Predicts Systemic Thromboembolic Events in Elderly After Cardiac Electronic Device Implantation
Source: Front Med (Lausanne). 2021 Dec 24;8:786779. doi: 10.3389/fmed.2021.786779 (PMC8739510; doi:10.3389/fmed.2021.786779)
Supplement: Supplementary file 1 [file Data_Sheet_1.PDF]

SUPPLEMENT TABLE 1 | Baseline characteristics of the overall study group and with/without new onset atrial fibrillation

	**All Patients (N = 219)**	**New onset atrial fibrillation**		**Univariate p**
		**Yes (N = 22)**	**No (N = 197)**	
Age (years)	77 (71–84)	82 (74–84)	77 (71–84)	0.340
Sex				0.039
Male	134 (61.2%)	18 (81.8%)	116 (58.9%)	
Female	85 (38.8%)	4 (18.2%)	81 (41.1%)	
BMI (kg/m^2)	24.2 (22.2–25.9)	24.1 (22.2–25.8)	24.3 (22.2–25.9)	0.793
Device type				0.228
Dual chamber PM	173 (79.0%)	21 (95.5%)	152 (77.2%)	
Dual chamber ICD	23 (10.5%)	0 (0.0%)	23 (11.7%)	
CRTP	19 (8.7%)	1 (4.5%)	18 (9.1%)	
CRTD	4 (1.8%)	0 (0.0%)	4 (2.0%)	
Primary Indication				0.191
Sinus node dysfunction	118 (53.9%)	13 (59.1%)	128 (65.0%)	
Atrioventricular block	55 (25.1%)	8 (36.4%)	47 (23.9%)	

Heart failure/VT/VF	46 (21.0%)	1 (4.5%)	22 (11.2%)	
Atrial pacing (%)	33.5 (9.1–79.1)	39.8 (13.9–64.6)	32.9 (8.6–79.8)	0.886
Ventricular pacing (%)	4.3 (0.2–98.7)	14.4 (0.3–36.5)	2.9 (0.2–98.8)	0.755
Hypertension	202 (92.2%)	21 (95.5%)	181 (91.9%)	1.000
Diabetes mellitus	114 (52.1%)	13 (59.1%)	101 (51.3%)	0.486
Hyperlipidemia	189 (86.3%)	22 (100.0%)	167 (84.8%)	0.050
Chronic obstructive pulmonary disease	14 (6.4%)	3 (13.6%)	11 (5.6%)	0.154
Prior myocardial infarction	46 (21.0%)	6 (27.3%)	40 (20.3%)	0.447
Coronary artery disease	67 (30.6%)	7 (31.8%)	60 (30.5%)	0.895
Heart failure				0.398
Preserved LVEF	24 (11.0%)	4 (18.2%)	20 (10.2%)	
Reduced LVEF	48 (21.9%)	5 (22.7%)	43 (21.8%)	
None	147 (67.1%)	13 (59.1%)	134 (68.0%)	
Chronic kidney disease	87 (39.7%)	12 (54.5%)	75 (38.1%)	0.134
Chronic liver disease	8 (3.7%)	0 (0.0%)	8 (4.1%)	1.000
Thyroid disease	16 (7.3%)	0 (0.0%)	16 (8.1%)	1.000
Peripheral artery disease	4 (1.8%)	1 (4.5%)	3 (1.5%)	0.347
Valvular heart disease	25 (11.4%)	2 (9.1%)	23 (11.7%)	1.000

Hemoglobin (mg/dL)	12.0 (10.8–13.0)	11.3 (10.0–12.0)	12.0 (10.7–13.0)	0.695
Echo parameters				
LVEF (%)	66.7 (54.0–73.0)	62.5 (51.0–70.5)	67.0 (55.0–73.5)	0.369
Mitral E/e'	11.8 (9.0–14.0)	12.0 (8.2–15.0)	11.7 (9.0–14.0)	0.819
LA diameter (cm)	3.8 (3.4–4.1)	4.1 (3.5–4.4)	3.8 (3.3–4.1)	0.110
RV systolic function (s', m/s)	12.0 (11.0–14.0)	12.0 (11.8–14.0)	12.0 (11.0–14.0)	0.194
Drugs prescribed at baseline				
Antiplatelets	92 (42.0%)	6 (27.3%)	86 (43.7%)	0.140
Anticoagulants	21 (9.6%)	12 (54.5%)	9 (4.6%)	<0.001
Beta blockers	72 (32.9%)	10 (45.5%)	62 (31.5%)	0.185
Ivabradine	16 (7.3%)	2 (9.1%)	14 (7.1%)	0.667
Amiodarone	37 (16.9%)	8 (36.4%)	29 (14.7%)	0.010
Flecainide	1 (0.5%)	0 (0.0%)	1 (0.5%)	1.000
Propafenone	8 (3.7%)	2 (9.1%)	6 (3.0%)	0.186
Digoxin	4 (1.8%)	0 (0.0%)	4 (2.0%)	1.000
non-DHP CCBs	6 (2.7%)	0 (0.0%)	6 (3.0%)	1.000
RAAS inhibitors	104 (47.7%)	9 (40.9%)	95 (48.5%)	0.501

Diuretics	34 (15.5%)	7 (31.8%)	27 (13.7%)	0.026
Statins	90 (41.1%)	8 (36.4%)	82 (41.6%)	0.634
Metformin	38 (17.4%)	5 (22.7%)	33 (16.8%)	0.483
SGLT2 inhibitors	9 (4.1%)	1 (4.5%)	8 (4.1%)	1.000
CHA_2DS_2-VASc score	4 (3–4)	4 (3–4)	4 (3–4)	0.707
C_2HEST score	3 (3–4)	3 (3–4)	3 (2–4)	0.172
mC_2HEST score	3 (3–4)	3 (3–4)	3 (3–4)	0.147
HAVOC score	4 (4–8)	6 (4–8)	4 (4–8)	0.204
HAT_2CH_2 score	2 (2–3)	3 (2–4)	2 (2–3)	0.025
AHRE ≥ 24hrs	27 (12.3%)	11 (50.0%)	16 (8.1%)	<0.001

Data are presented as the median (interquartile interval) or n (%). Non-parametric continuous variables, as assessed using the Kolmogorov–Smirnov method, were analyzed using the Mann–Whitney U test. Statistical significance is set at $p < 0.05$.

BMI, body mass index; PM, pacemaker; ICD, implantable cardioverter defibrillator; CRTP, cardiac resynchronization therapy pacemaker; CRTD, cardiac resynchronization therapy defibrillator; VT, ventricular tachycardia; VF, ventricular fibrillation; LVEF, left ventricular ejection fraction; LA, left atrium; RV, right ventricle; non-DHP CCBs, non-dihydropyridine calcium channel blockers; RAAS, renin-angiotensin-aldosterone system; SGLT2, sodium glucose co-transporters 2; CHA_2DS_2-Vasc score: Range from 0 to 9. History of heart failure, hypertension, diabetes, vascular disease, age 65–74 years, and female sex each is calculated as 1 point; 75

years or older and prior stroke, TIA, or thromboembolism each is calculated as 2 points; C_2HEST score: Range from 0 to 8. C_2: CAD/COPD (1 point each); H: hypertension (1 point); E: elderly (age ≥ 75 years, 2 points); S: systolic HF (2 points); and T: thyroid disease (hyperthyroidism, 1 point); mC_2HEST score: Range from 0 to 8. C_2: CAD/COPD (1 point each); H: hypertension (1 point); E: elderly (age 65~74 years, 1 point; age ≥ 75 years, 2 points); S: systolic HF (2 points); and T: thyroid disease (hyperthyroidism, 1 point); HAVOC score: H: hypertension (2 points); A: age (age ≥ 75 years, 2 points); V: valvular heart disease (2 points), peripheral vascular disease (1 point); O: obesity (1 point); C: congestive heart failure (4 points) and coronary artery disease (2 points); HAT_2CH_2 score: Range from 0 to 7. Hypertension, 1 point; age >75 years, 1 point; stroke or transient ischemic attack, 2 points; chronic obstructive pulmonary disease, 1 point; heart failure, 2 points; AHRE, atrial high-rate episodes
